# Twist1 Regulates the Immune Checkpoint VISTA and Promotes the Proliferation, Migration and Progression of Pancreatic Cancer Cells

**DOI:** 10.1111/jcmm.70586

**Published:** 2025-05-08

**Authors:** Kubra Sena Bas Topcu, Ercan Cacan

**Affiliations:** ^1^ Department of Molecular Biology and Genetics, Faculty of Science Bartin University Bartin Türkiye; ^2^ Department of Molecular Biology and Genetics, Faculty of art and Science Tokat Gaziosmanpasa University Tokat Türkiye

**Keywords:** EMT, immune checkpoint, pancreatic cancer, Twist1, vorinostat

## Abstract

Pancreatic cancer is one of the deadliest malignant tumours worldwide. Despite the developments in the treatments of pancreatic cancer, survival rates remain at a low level, and the mechanisms underlying the aggressive course of the cancer are not fully understood. VISTA is an immune checkpoint and has recently become a significant target in cancer treatment; however, the roles of VISTA in the development of pancreatic cancer have largely remained unknown. Histone deacetylase inhibitors (HDACi) have been reported to reverse the epithelial‐mesenchymal transition (EMT) and may enhance the efficacy of anti‐PD‐1 therapy. The PD‐L1/PD‐1 immune checkpoint targeted by this therapy shares structural similarity with VISTA. Moreover, combination therapy of vorinostat and anti‐PD‐1 has been shown to significantly reduce tumour growth by suppressing the transcription factor c‐Myc. Therefore, in this study, we aim to investigate the genes that are associated with EMT and explore the potential mechanism involving Twist1, a proto‐oncogene, and VISTA in pancreatic cancer. We also sought to determine the synergistic effects of an HDACi, vorinostat, in combination with Twist1‐siRNA on VISTA expression in pancreatic cancer cells’ viability and proliferation. Our results revealed that Twist1 blockade in combination with vorinostat in pancreatic cancer cells suppresses EMT‐associated genes and the immune checkpoint VISTA compared to treatments administered alone. As a result, identifying the genes associated with EMT in pancreatic cancer and understanding the role of Twist1 in this process is a crucial step to contribute to the identification of new targets for pancreatic cancer treatment and the improvement of existing treatment strategies.

## 1 Introduction

Pancreatic cancer is one of the main causes of cancer‐related deaths worldwide. In the last 25 years, the global incidence of pancreatic cancer has surged by more than two‐fold and the 5‐year survival rate is about 11%, despite advancements in cancer treatment [1, 2]. The low survival rate is due to late diagnosis, limited treatment options, and resistance mechanisms of tumours. Although surgery and chemotherapy are the main treatment options, targeted therapies and immunotherapy offer new hopes for patients [3, 4, 5, 6]. Therefore, the investigation of molecular mechanisms of pancreatic cancer and the discovery of new biomarkers are of critical importance.

Immune checkpoints, which are highly expressed in the development of cancer, suppress the immune response and promote the proliferation of tumour cells. In recent years, treatment methods targeting immune checkpoints have made significant progress in cancer treatment [7, 8]. However, the efficacy of immune checkpoint inhibitors is limited, and investigating their synergistic effects with combination therapies is important to develop more successful treatment strategies [9]. Development of such agents and their combination with existing treatments may increase therapeutic synergy [10, 11]. The V‐domain Ig suppressor of T‐cell activation (VISTA, PD‐1H, Gi24, Dies‐1, DD1α) is a newly identified immune checkpoint and a member of the B7/CD28 family and is highly expressed in the tumour microenvironment (TME) [10, 12, 13]. VISTA protein has been shown to have immune inhibitory properties in many cancer types, including melanoma, prostate, kidney, lung, colon, brain, ovarian, endometrial cancers, and acute myeloid leukaemia [14]. VISTA, which functions as both a ligand and a receptor in immune regulation, is also known to shape immune responses. Ligands that interact with VISTA have been found to be expressed in tumour cells and directly suppress T cell activity [15]. Studies have shown that human peripheral monocytes produce soluble VISTA, which inhibits the cytotoxic activity of T cells but does not initiate apoptosis [16]. VISTA expression is highly expressed in tumour‐infiltrating T cells and myeloid cells in human cancer tissues. In many cancer types, low patient survival and treatment resistance have been associated with high VISTA expression [17, 18]. Furthermore, VISTA expression is regulated by TGF‐β, a transcription factor that induces epithelial‐mesenchymal transition (EMT). TGF‐β increases VISTA expression by activating Smad3, but this effect is observed only in non‐cytotoxic cells in T lymphocytes. It is thought that this differential effect of TGF‐β may be related to chromatin reorganisation [19, 20, 21]. High VISTA expression has been shown to be associated with EMT in breast cancer [14]. For these reasons, studying the expression of immune checkpoint mediators and their relationships with epigenetic regulators is critical for understanding immune signalling pathways and selecting effective immunotherapies [19, 20, 21, 22]. Elucidation of the epigenetic mechanisms regulating VISTA expression and development of combination therapies with HDAC inhibitors may offer new approaches to cancer treatment.

EMT is known as an important initiator of tumour invasion and metastasis and has been shown to be associated with poor prognosis and chemotherapy resistance in pancreatic cancer [23, 24]. Twist1, a fundamental helix–loop–helix (bHLH) transcription factor that plays a crucial role in embryonic development and morphogenesis, is highly expressed in various tumour types and serves as a significant regulator of cancer metastasis [25, 26]. Additionally, Twist1 has been identified to correlate with tumorigenesis, angiogenesis, and poor prognosis in various human cancers [27, 28, 29, 30]. EMT‐associated transcription factors lead to PD‐L1 upregulation in circulating tumour cells and promote the infiltration of immune cells into the tumour microenvironment. In addition, EMT‐regulating transcription factors promote tumour invasion and metastasis by increasing immune checkpoint expression [31]. Twist1 has been shown to be regulated by the immune checkpoint PD‐L1 expression in breast cancer cells [32]. Although Twist1 is known to facilitate tumour progression by suppressing the immune system, there are no studies examining the relationship between Twist1 and the immune checkpoint VISTA.

Vorinostat (SAHA) is an HDAC inhibitor that interferes with cell growth and survival by increasing overall gene transcription. Vorinostat is known as the first HDAC inhibitor approved by the FDA for the treatment of lymphoma in 2006. In addition to conventional cancer treatments, strategies combining epigenetic modifiers with immunotherapy, chemotherapy, or ionising radiation have been explored in cell line models and clinical trials, showing promise for cancer treatment [33, 34, 35, 36, 37, 38, 39]. Studies have shown that treatment with vorinostat significantly inhibits cell growth and promotes apoptosis in various types of cancer [40, 41, 42]. In addition, vorinostat treatment reversed the change in EMT in pancreatic cancer cells and was found to reduce the invasion of cancer cells [43]. In a study conducted by Wu et al. (2020) on melanoma, the effects of vorinostat treatment on PD‐L1, an immune checkpoint structurally similar to VISTA, were examined. In their study, they found that the combination of vorinostat and anti‐PD‐1 treatment significantly reduced tumour growth by suppressing the transcription factor c‐Myc [44]. It has been reported that HDAC5 modulates PD‐L1 expression and cancer immunity via p65 deacetylation in pancreatic cancer, and vorinostat decreases PD‐L1 expression in the triple‐negative breast cancer cell line [45, 46]. However, the effects of HDAC inhibitors on EMT are complex and vary depending on the type of cancer. It has been shown that HDAC inhibitors can suppress cell migration while increasing vimentin expression in cholangiocellular carcinoma [47]. A recent study has shown that increased Twist1 expression causes drug resistance to HDAC inhibitors in synovial sarcoma cells [48]. These findings highlight the need to further investigate the therapeutic potential of HDAC inhibitors in cancer treatment. In particular, the combination of HDAC inhibitors with strategies that suppress EMT may overcome resistance and increase the efficacy of current treatments.

The molecular mechanisms regulating the expression of the immune checkpoint VISTA in pancreatic cancer, its role in invasion and metastasis, and its relationship with EMT are not yet fully understood. To address these deficiencies, our study focuses on the role of EMT in tumour progression and immune regulation. In particular, the effects of the transcription factor Twist1 and the HDAC inhibitor vorinostat on VISTA were investigated. The effects of these two factors on cell viability, migration, and EMT‐related gene expression in pancreatic cancer cells were also evaluated.

## 2 Material and Method

### 2.1 Expression EMT‐Related Genes and VISTA in Pancreatic Cancer

The Gene Expression Profiling Interactive Analysis 2 (GEPIA2) database (http://gepia2.cancer‐pku.cn/) is an online database that allows gene expression analysis based on tumour and normal samples from The Cancer Genome Atlas (TCGA) dataset and Genotype‐Tissue Expression (GTEx) databases [49, 50]. The study involved an interactive analysis of gene expression profiling using TCGA and GTEx databases through the GEPIA2 database. The expression levels of Twist1, TGF‐β, E‐Cadherin, N‐Cadherin, miR‐21, Snail, Slug, Vimentin, and VISTA genes were investigated. The data obtained from TCGA encompassed 171 normal control tissue samples and 179 pancreatic cancer (PAAD) samples. In the GEPIA2 database, under the “Expression DIY” tab, settings were chosen with a *p* value cutoff of 0.05 and a log2FC cutoff of 0.1.

### 2.2 Cell Culture and Reagents

Vorinostat was purchased from Ambeed, Twist1‐siRNA (5 nmol), non‐target siRNA (NT‐siRNA, 5 nmol), and Lipofectamine 3000 were purchased from Invitrogen. Primers for the TGF‐β, E‐cadherin, N‐cadherin, miR‐21, snail, slug, vimentin, GAPDH and VISTA genes were purchased from Macrogen, while primers for the Twist1 gene were obtained from Sentebiolab.

Rabbit anti‐human antibody VISTA was purchased from Cell Signalling (Danvers, MA, USA), anti‐rabbit secondary antibody was purchased from Santa Cruz and β‐actin antibody [HRP] was obtained from GenScript. AsPC‐1 (ATCC, CRL‐1682), Mia PaCa‐2 (ATCC, CRL‐1420) and CAPAN‐1 (ATCC, HTB‐79) pancreatic cancer cells were incubated in a medium containing 10% fetal bovine serum (FBS; Biowest, France) and 1% penicillin–streptomycin. AsPC‐1 cells were incubated in RPMI 1640 medium (Biowest, France), while Mia PaCa‐2 and CAPAN‐1 cells were incubated in DMEM medium (Gibco, USA) at 37°C in a humidified incubator with 5% CO_2_.

### 2.3 Cell Transfection

Pancreatic cancer cells were treated with Twist1‐siRNA to silence the Twist1 gene. The siRNA was prepared in a serum‐free and antibiotic‐free medium, and transfection was carried out using Lipofectamine 3000 according to the manufacturer's protocol for both NT‐siRNA and Twist1‐siRNA.

### 2.4 Cell Viability Analysis

Cell viability was assessed using MTT assays. A total of 5000 cells were seeded into each well in a 96‐well plate and incubated for 24 h. The next day, the old medium was removed, and fresh medium with different concentrations of vorinostat (0.1–200 μM) was applied to the pancreatic cancer cells and incubated for an additional 48 h. Twist1‐siRNA and NT‐siRNA (Thermo Fisher Scientific) were prepared at a concentration of 3 pmol and placed in an incubator at 37°C with 5% CO_2_ for 48 h. A 5 mg/mL MTT solution was prepared by dissolving it in PBS, and 20 μL of the MTT solution was added. The samples were then incubated in a 37°C incubator with 5% CO_2_ for 3 h. The MTT solution was then discarded, and 100 μL of DMSO was added. The absorbance value was read at 575 nm using a microplate reader [51].

### 2.5 Cell Migration

A wound healing assay was conducted to assess the impact of Twist1 suppression and vorinostat treatment on pancreatic cancer cell migration. Mia PaCa‐2, AsPC‐1 and CAPAN‐1 cells were plated in 6‐well plates at a density of 600,000 cells per well and incubated at 37°C under 5% humidified CO_2_ until reaching 80%–90% confluence. Subsequently, a wound area was manually created in the middle of each well using a 200 μL pipette tip. The old medium was then removed, and the cells were washed with PBS to eliminate any remaining cell fragments on the surface. The IC_50_ dose of Twist1‐siRNA and vorinostat was added to each 6‐well plate. Subsequent to the treatment, images of migration areas were captured at 0, 24 and 48 h, and the images were analysed using Image J [52].

### 2.6 Quantitative RT‐PCR

Pancreatic cancer cells were seeded into 6‐well plates for qRT‐PCR analysis. Each well contained 1,000,000 cells, and the cells were incubated for 24 h. After the incubation period, the old medium was removed, and the cells were treated with 3 pmol of Twist1‐siRNA, 3 pmol NT‐siRNA, and vorinostat at the IC_50_ concentrations separately or in combination for each cell line. This was followed by a 48‐h incubation period. After incubation, the cells were collected for RNA isolation, which was carried out using the Hibrizol reagent (Hibrigen, Turkiye) following the manufacturer's protocol. Gene expression levels were determined through quantitative real‐time polymerase chain reaction (qRT‐PCR) using the ‘Luna Universal One‐Step RT‐qPCR Kit. The primers used in this study are presented in Table 1.

**TABLE 1:** Reverse transcription‐quantitative PCR primer sequences.

Gene	Primer sequence
Homo Twist1	F: 5′ TCC GCA GTC TTA CGA GGA GCT ‘3 R: 5′ TCT GAA TCT TGC TCA GCT TGT CCG ‘3
Homo E Cadherin	F: 5′ ATT TTT CCC TCG ACA CCC GAT ‘3 R: 5′ TCC CAG GCG TAG ACC AAG A ‘3
Homo N Cadherin	F: 5′ CCT CCA GAG TTT ACT GCC ATG AC ‘3 R: 5′ GTA GGA TCT CCG CCA CTG ATT C ‘3
Homo Vimentin	F: 5′ ACG TCT TGA CCT TGA ACG CA ‘3 R: 5′ TCT TGG CAG CCA CAC TTT CA ‘3
Homo Snail	F: 5′ TGC CCT CAA GAT GCA CAT CCG A ‘3 R: 5′ GGG ACA GGA AGA AGG GCT TCT C ‘3
Homo Slug	F: 5′ ATC TGC GGC AAG GCG TTT TCC A ‘3 R: 5′ GAG CCC TCA GAT TTG ACC TGT C ‘3
Homo TGF‐β	F: 5′ GCC AGA GTG GTT ATC TTT TGA TG ‘3 R: 5′ AGT GTG TTA TCC CTG CTG TCA C ‘3
Homo miR‐21	F: 5′ GCG GCG TAG CTT ATC AGA CTG A ‘3 R: 5′ GTG CAG GGT CCG AGG T ‘3
Homo VISTA	F: 5′ GAT AGC GGC CTC TAC TGC TG ‘3 R: 5′ TGG ATG GTG CAT CTT TGC CT ‘3
Homo GAPDH	F: 5′ AAG GTG AAG GTC GGA GTC AA ‘3 R: 5′ AAT GAA GGG GTC ATT GAT GG ‘3

GAPDH was used as the reference gene, and calculations were performed by using the 2^(−ΔΔCt)^ method [53]. The fold change in expression was calculated from three independent experiments.

### 2.7 Western Blot Analysis

Pancreatic cancer cells were seeded in a 6‐well plate, with 1,000,000 cells in each well, and incubated until reaching approximately 80% confluence. After the incubation period, the old medium was removed, and five different groups were established: the control group, the group treated with vorinostat IC_50_ dose, Twist1‐siRNA 3 pmol group, NT‐siRNA 3 pmol group, and the group treated with vorinostat IC_50_ dose + Twist1‐siRNA 3 pmol. Cells were then incubated for 72 h and washed twice with cold PBS. Subsequently, the cells were scraped using a scraper, collected in eppendorf tubes, and centrifuged at 2000 rpm for 5 min. The resulting pellet dissolved in 200 μL of RIPA buffer. The cell lysis was centrifugate at 12,000 rpm for 30 min at + 4°C and the supernatant was transferred to new tubes. The protein levels of the samples were measured using the “Pierce BCA Protein Assay Kit” (Thermo Scientific). SDS‐PAGE electrophoresis was performed, and the samples were transferred to a 0.45 μm nitrocellulose membrane using the Bio‐Rad Trans‐Blot Turbo Transfer System. Subsequently, the membranes were blocked with 5% milk powder at room temperature for 1 h and washed with 1 × TBS/T for 5 min. The membranes were then incubated with the primary antibody overnight at 4°C. Following another wash with TBS‐T, the membranes were treated with an HRP‐conjugated secondary antibody for 1 h at room temperature. Finally, the membranes treated with the ECL solution (Bio‐Rad, Clarity ECL Western Blotting Substrate, USA) were imaged using the Fusion FX‐7 system (Vilber, Germany) at 4°C. Blot analysis was conducted using the ImageJ program [54].

### 2.8 Statistical Analysis

Statistical analyses were conducted using GraphPad Prism V.9.5.1 (GraphPad Software). When the data exhibited a normal distribution, group means were compared using the Student's *t*‐test with a two‐tailed distribution. qRT‐PCR data were presented as mean ± SEM. Two‐way ANOVA was employed to compare differences between groups. Significance was assessed by calculating *p* values, and a threshold of *p* < 0.05 was considered statistically significant.

(****: *p* < 0.0001; ***: *p* < 0.001; **: *p* < 0.01; *: *p* < 0.05).

## 3 Results

### 3.1 Expression of Genes Associated With EMT and the Immune Checkpoint VISTA in Pancreatic Cancer Tissue

**FIGURE 1:**
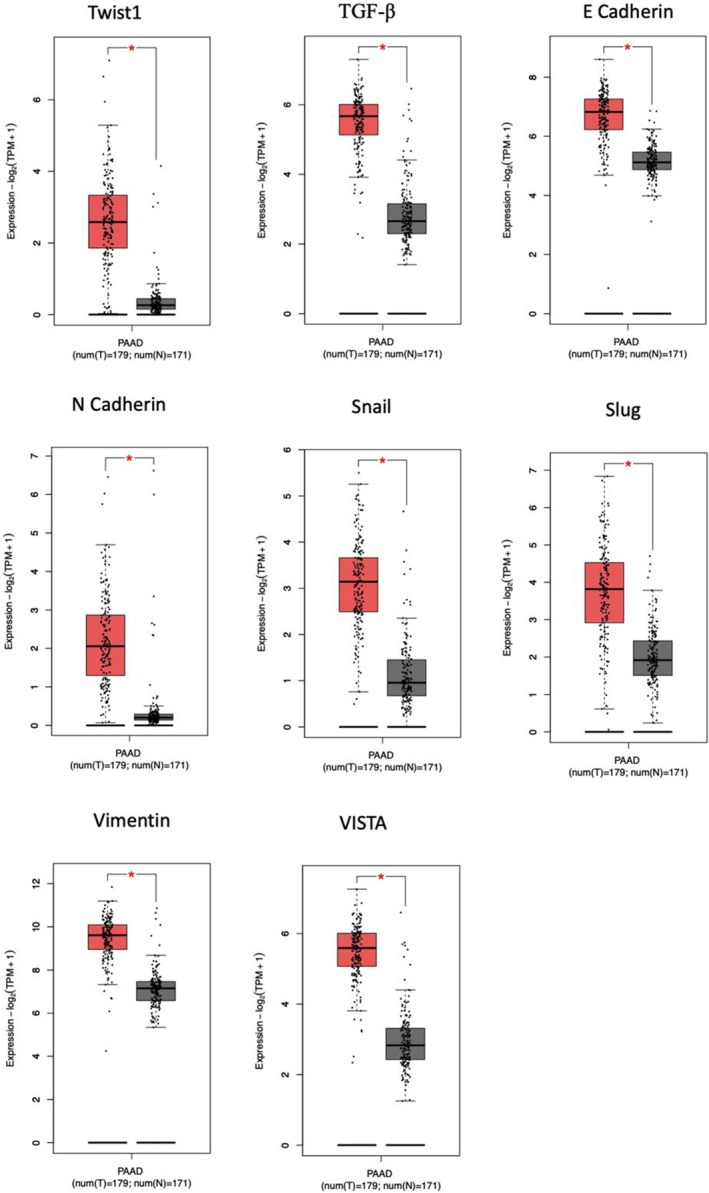
Differential expression analysis of target genes in pancreatic adenocarcinoma (PRAD) samples (red, T = 179) and normal tissue (grey, *N* = 171) from the TCGA and GTEx projects was performed with GEPIA2. Gene expression (log2 (TPM + 1)) for Twist1, TGF‐β, E Cadherin, N Cadherin, Snail, Slug, Vimentin and VISTA. *p value ≤ 0.05.

The expression levels of Twist1, TGF‐β, E‐cadherin, N‐cadherin, miR‐21, snail, slug, vimentin and VISTA genes were examined in pancreatic adenocarcinoma (PAAD) and normal pancreatic tissues by using the GEPIA2 database. The data were evaluated using TCGA and GTEx pan‐cancer datasets. Analysis of TCGA/GTEx data in GEPIA2 showed that Twist1, TGF‐β, E‐Cadherin, N‐Cadherin, Snail, Slug, Vimentin and VISTA expressions were significantly upregulated in PAAD samples compared to healthy pancreatic tissues (Figure 1). This observation indicates a potential role for the target genes in the molecular mechanism of pancreatic adenocarcinoma, suggesting that their downregulation may contribute to the pathogenesis of the cancer. Further exploration of these findings could provide valuable insights into the molecular mechanisms underlying pancreatic adenocarcinoma and potential avenues for targeted therapeutic interventions. Previous studies have investigated the potential effects of vorinostat on EMT [43, 55]. In this study, we comprehensively examined the link between vorinostat and EMT and how its combined treatment with Twist1 inhibition affected the expression levels of immune checkpoint VISTA and EMT‐related genes.

### 3.2 Combination Treatment of Twist1‐siRNA Transfection and Vorinostat Inhibits Cell Migration and Proliferation in Pancreatic Cancer

**FIGURE 2:**
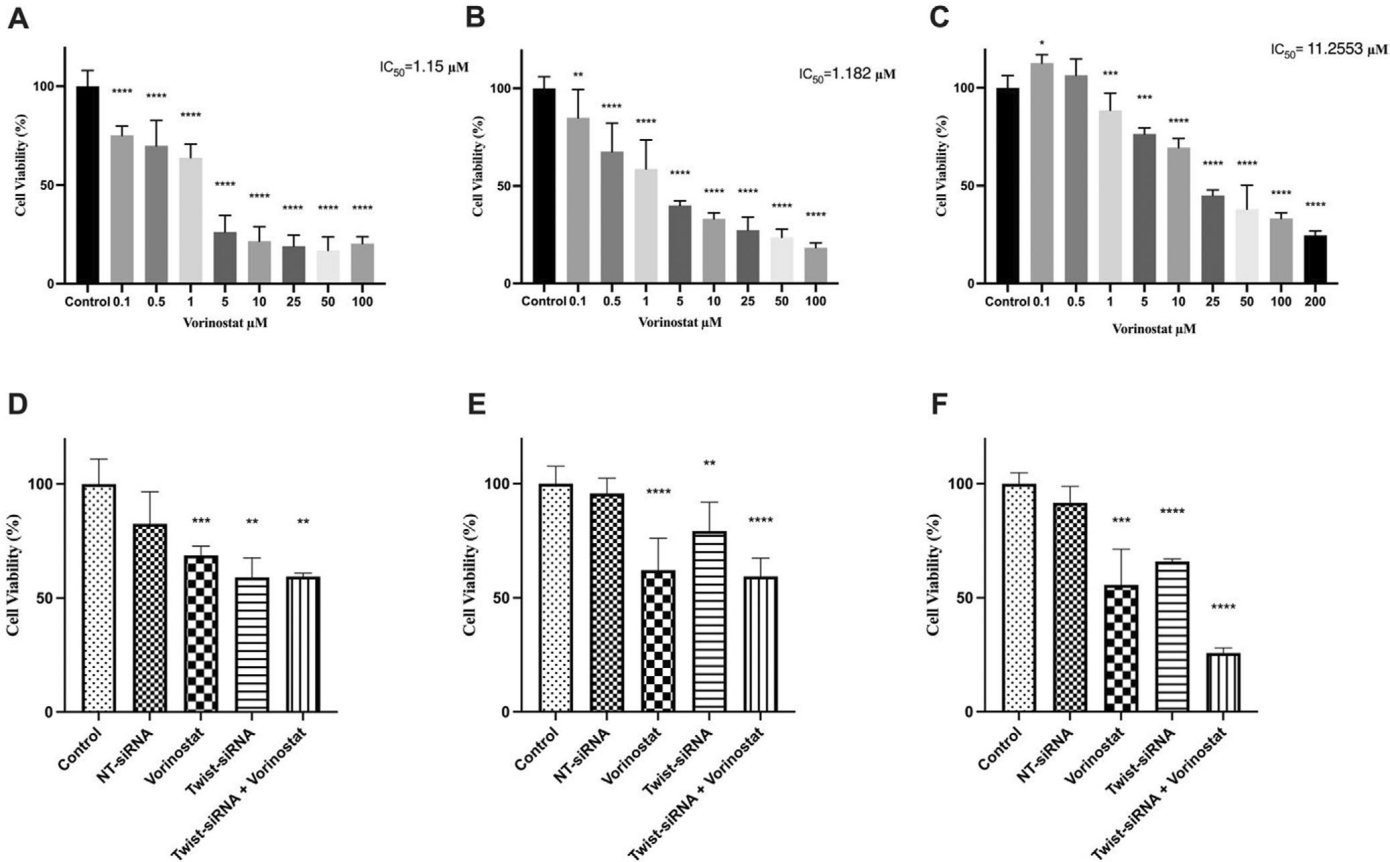
The effect of Twist1‐siRNA, vorinostat and combination treatment on cell viability was evaluated by MTT assays. The results were compared to untreated pancreatic cancer cells. Experiments were performed in triplicate. Application of vorinostat for 48 h inhibited the viability of Mia PaCa‐2 (A), AsPC‐1 (B) and CAPAN‐1 (C) human pancreatic cancer cell lines in a dose‐dependent manner. Combination treatment with vorinostat was also performed following the Twist1‐siRNA and NT‐siRNA. The viability of cells following the siRNA and combination treatment in Mia PaCa‐2 (D), AsPC‐1 (E) and CAPAN‐1 (F) cell lines was significantly decreased compared to single‐agent treatment.

To assess the cytotoxic effects of the HDAC inhibitor vorinostat, pancreatic cancer cell lines were exposed to various concentrations of vorinostat for 48 h. The results revealed a decrease in cell viability in all three pancreatic cancer cell lines at lower vorinostat concentrations within 48 h (Figure 2). The IC_50_ values for vorinostat were determined as 1.15 μM for the Mia PaCa‐2 cell line, 1.18 μM for the AsPC‐1 cell line, and 11.26 μM for the CAPAN‐1 cell line. These IC_50_ values were subsequently employed in further investigations.

To investigate the cytotoxic effects of Twist1 gene knockdown in pancreatic cancer cell lines, Twist1‐siRNA and NT‐siRNA were utilised. Following the protocol provided by the manufacturer, 3 pmol of siRNA was transfected into the cells. After determining the IC_50_ values through MTT analysis, the viability of pancreatic cancer cells was examined following the combined application of vorinostat and Twist1‐siRNA. To achieve this, the effective dose of vorinostat, in combination with 3 pmol of Twist1‐siRNA, was administered to pancreatic cancer cell lines for 48 h. The combination of Twist1‐siRNA and vorinostat given to pancreatic cancer cell lines has significantly reduced cell viability in CAPAN‐1 cells compared to other cells when used alone (Figure 2).

**FIGURE 3:**
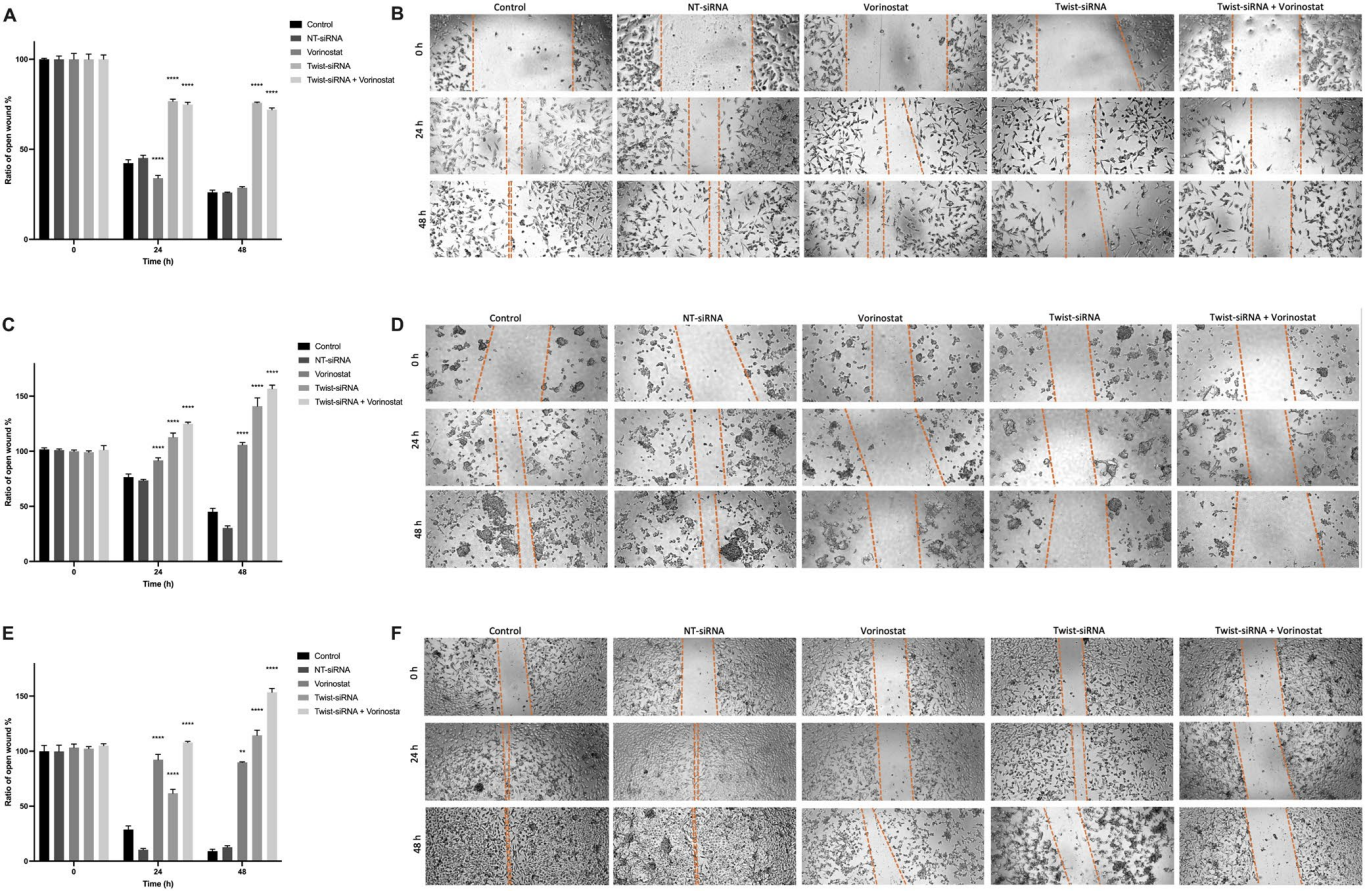
Wound healing assay was performed to observe cell migration in pancreatic cancer cell lines. Cell migration was evaluated by microscopic images. The ratio of open wound percent and the microscope images of Mia PaCa‐2 (A, B), AsPC‐1 (C, D) and CAPAN‐1 (E, F) cell lines obtained at 5× magnification.

Cell migration analysis was performed to assess the effects of Twist1‐siRNA transfection and vorinostat treatment on cell migration. The combination of vorinostat and Twist1‐siRNA in CAPAN‐1 and AsPC‐1 cell lines more effectively inhibited cell migration compared to their individual treatments (Figure 3A–F). These findings highlight the potential therapeutic benefit of the combination of Twist1 blockade with vorinostat in the treatment of pancreatic cancer, clearly showing that it has a stronger effect in suppressing cell migration.

### 3.3 Vorinostat Treatment Increases the Expression of EMT‐Related Genes and VISTA

**FIGURE 4:**
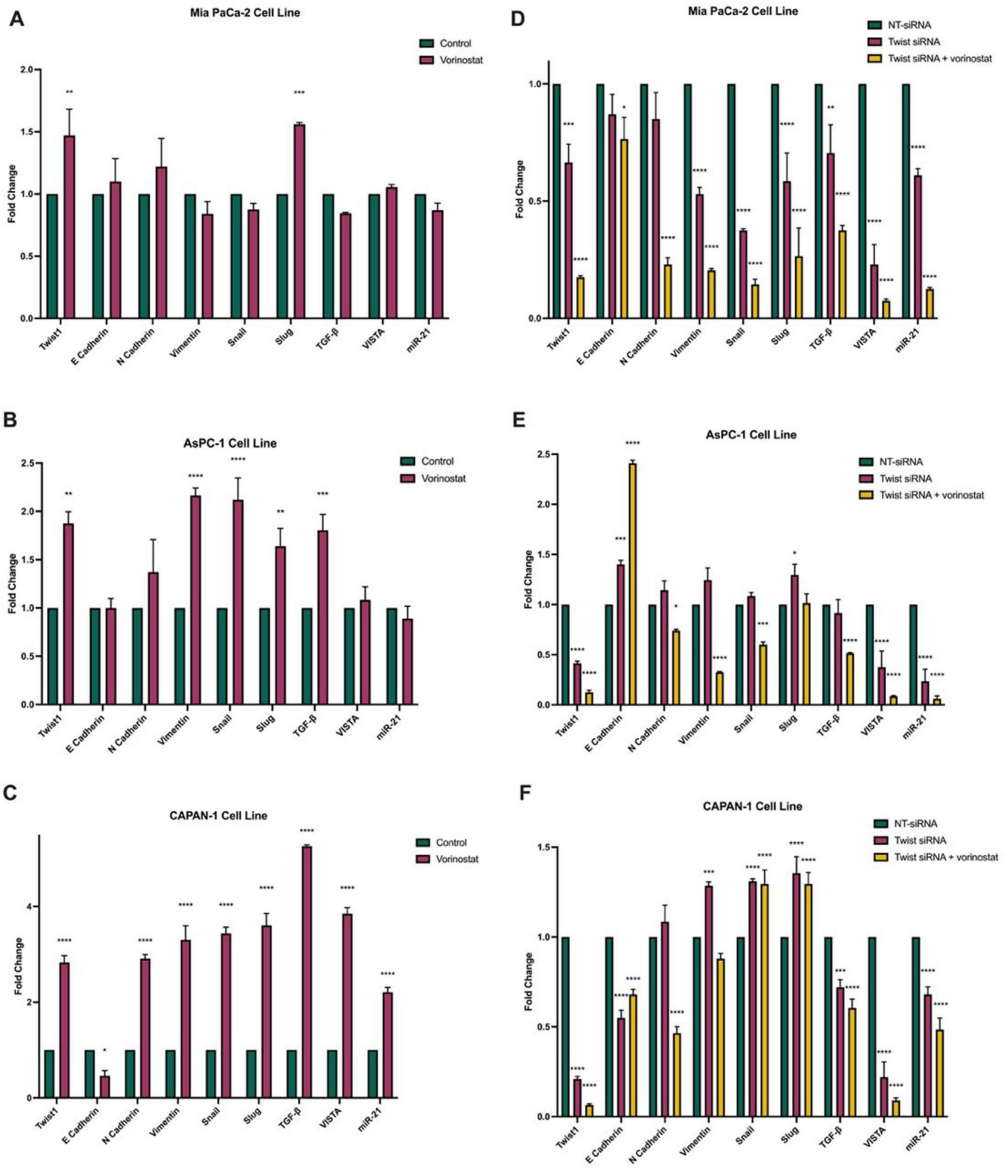
Expression levels of target genes in pancreatic cancer cell lines following the treatment of the HDAC inhibitor, vorinostat (A–C), and Twist1‐siRNA (D–F) separately or in combination.

To further investigate the effects of vorinostat on pancreatic cancer, a low dose of vorinostat (IC_50_ dose) was administered to pancreatic cancer cell lines for 48 h. Expression levels of EMT‐related genes and VISTA were examined. The results revealed an increase in the expression levels of EMT‐related genes in CAPAN‐1 (11.25 μM vorinostat) and AsPC‐1 (1.18 μM vorinostat) cell lines compared to the non‐vorinostat group. Specifically, vorinostat treatment significantly upregulated Twist1 gene expression in all three pancreatic cancer cell lines. Moreover, vorinostat treatment in the CAPAN‐1 cell line led to a notable 3.5‐fold increase in the expression of the immune checkpoint VISTA compared to the control group (Figure 4A–C). These findings suggest that vorinostat, even at low doses, may influence EMT‐related genes and enhance VISTA expression, potentially implicating its role in pancreatic cancer progression and immune modulation.

### 3.4 The Transcription Factor Twist1 Regulates the Immune Checkpoint VISTA in Pancreatic Cancer

To further elucidate the role of Twist1 in modulating the immune checkpoint VISTA in pancreatic cancer, Twist1 knockdown was achieved through a 48‐h siRNA transfection in pancreatic cancer cell lines. Following the Twist1‐siRNA transfection, a noticeable decrease in the expression levels of EMT‐related genes was observed in Mia PaCa‐2 and AsPC‐1 pancreatic cancer cells. Furthermore, this knockdown significantly suppressed the expression level of the immune checkpoint VISTA in all three pancreatic cancer cell lines. Subsequently, a combination of vorinostat treatment and Twist1‐siRNA transfection was applied to pancreatic cancer cells for 48 h. The data demonstrate that, when compared with Twist1‐siRNA transfection alone, the combination with vorinostat more effectively suppressed the expression of EMT‐related genes and the immune checkpoint VISTA (Figure 4D–F). These results suggest a potential synergistic effect between Twist1 knockdown and vorinostat treatment in modulating both EMT‐related genes and the immune checkpoint VISTA in pancreatic cancer.

### 3.5 Combination of Twist1‐siRNA Transfection With Vorinostat Treatment of Pancreatic Cancer Cells Suppresses the Expression of the Immune Checkpoint VISTA Protein

**FIGURE 5:**
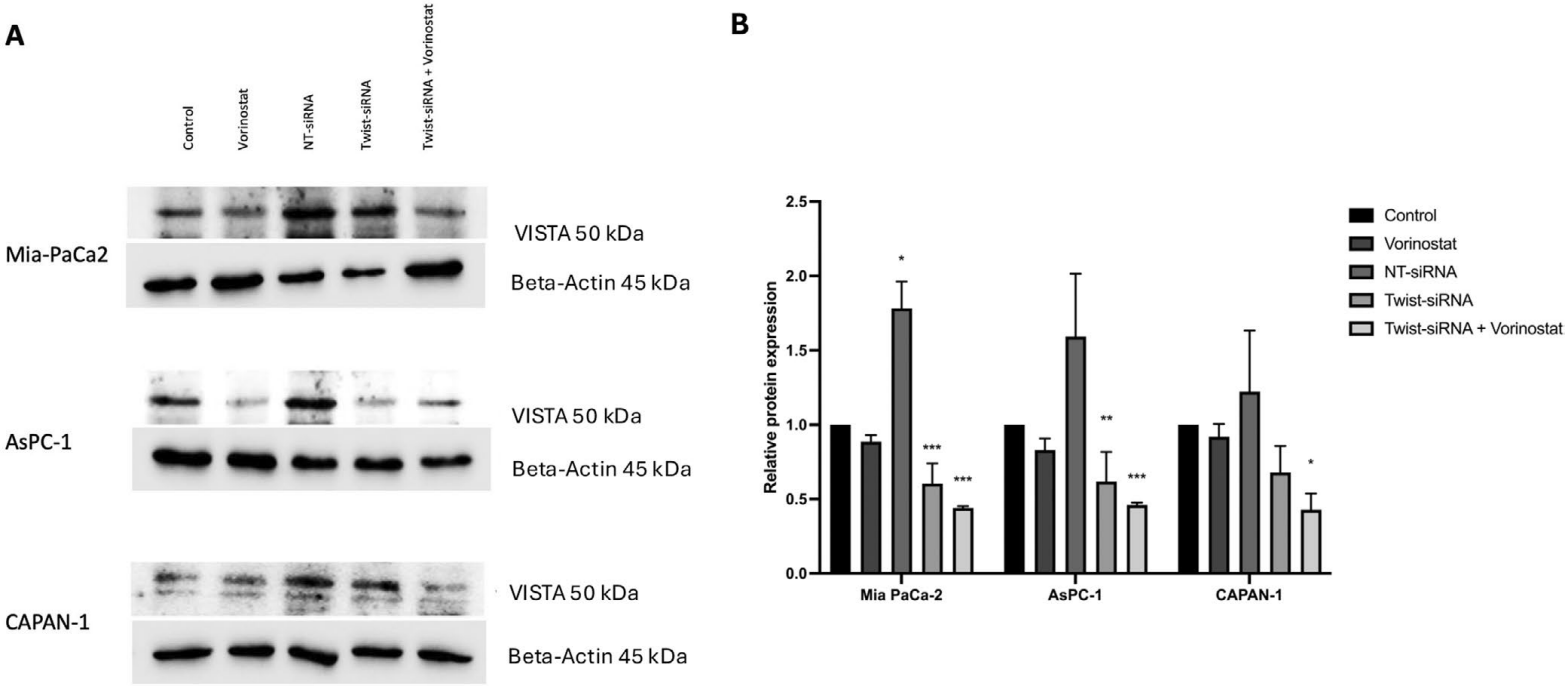
Western blot analysis was performed to detect VISTA protein expression level in AsPC‐1, Mia PaCa‐2, and CAPAN‐1 cells following the Twist1‐siRNA and vorinostat treatment. (A) The impact of siRNA transfection and vorinostat treatment, administered separately or in combination, on VISTA protein expression levels. (B) Changes in protein levels that were quantified by ImageJ software (version 1.53t).

To determine the effect of the HDAC inhibitor vorinostat, Twist1‐siRNA transfection, or combination treatment on the protein level of the immune checkpoint VISTA in pancreatic cancer cells, western blot analysis was performed. Our results showed no significant change in VISTA protein level following vorinostat treatment in pancreatic cancer cells. Compared to Twist1‐siRNA transfection, the combination of Twist1‐siRNA transfection and vorinostat treatment significantly decreased VISTA protein level. VISTA level decreased to approximately 20% following the Twist1‐siRNA transfection compared to the control group, and the combination treatment reduced VISTA level to approximately 50% (*p* < 0.001) particularly in the CAPAN‐1 cell line (Figure 5). The combination of siRNA targeting Twist1 and the HDAC inhibitor, vorinostat, resulted in a significant reduction in VISTA levels, suggesting that Twist1 works synergistically with epigenetic regulatory mechanisms.

## 4 Discussion

Pancreatic cancer is one of the cancers with the highest mortality rates. The majority of patients develop metastatic pancreatic cancer due to a lack of biomarkers [38]. Therefore, the identification of new biomarkers for early diagnosis and the development of therapeutic strategies to prevent metastasis are crucial for improving the survival of patients with pancreatic cancer [2, 56].

Expression of the immune checkpoint VISTA is associated with poor prognosis in pancreatic, breast, bladder, colorectal, and ovarian cancers, and drugs targeting VISTA are currently being developed for cancer treatment [12, 57, 58]. In addition, high VISTA expression is thought to impair patient response to chemotherapy by inhibiting the immune response and promoting disease recurrence [59, 60]. Therefore, the blockade of immune checkpoints has recently gained significant interest in the treatment of malignancy. However, there is relatively less information available on the immune checkpoint VISTA in pancreatic cancer.

EMT, which plays an important role in cancer cell chemoresistance, is a complex process controlled by various transcriptional regulators such as Twist1, Snail, Slug and Zeb1 [61, 62]. Twist1 expression is abnormally high in many tumours, such as breast cancer, prostate cancer, and hepatocellular carcinoma. Twist1 plays a role in tumorigenesis, tumour progression, invasion and metastasis in cancer [63]. A study has shown that Twist1 regulates the self‐renewal and metastasis of cancer stem cells in breast cancer and renders cancer cells insensitive to chemotherapy [64]. In addition, Twist1 has been shown to be associated with cell proliferation, invasion and migration in pancreatic cancer [65]. Interestingly, studies have observed hypermethylation in the promoter region of Twist1 in pancreatic cancer [66]. Another study has shown that high expression of Twist1 in pancreatic cancer is associated with poor prognosis and that Twist1 may be an important regulator of the Warburg effect [28]. Therefore, targeted therapeutic approaches targeting Twist1 may help prevent critical processes such as tumour progression, metastasis, and chemotherapy resistance in pancreatic cancer.

HDAC inhibitors are compounds that have been shown to have anti‐cancer effects in clinical studies and induce epigenetic remodelling. Vorinostat (SAHA; suberoylanilide hydroxamic acid), an HDAC inhibitor, is approved by the FDA for the treatment of myelodysplastic syndrome and cutaneous T‐cell lymphoma. It also has the potential to be used in the treatment of pancreatic cancer [67, 68, 69]. Studies in lung and colon cancer have shown that novel HDAC inhibitors increase E‐cadherin expression, while decreasing vimentin and Slug expression. These regulatory effects suggest that they may significantly inhibit the migratory abilities of cancer cells through the suppression of EMT [70]. In addition, a 2021 study showed that the HDAC inhibitor vorinostat modulated the EMT phenotype, causing a decrease in N Cadherin and Vimentin gene/protein expressions, while increasing E Cadherin gene expression [71].

In this study, we investigated the effects of Twist1 and the HDAC inhibitor vorinostat on VISTA expression levels in pancreatic cancer. In the current study, we found that Twist1 is an important regulator of the immune checkpoint VISTA in pancreatic cancer. We first show that Twist1 and VISTA are highly expressed in pancreatic cancer, and we then focused on the effects of EMT, examining the role and mechanisms of Twist1 and HDAC inhibition on proliferation, migration, and the impact on VISTA in human pancreatic cancer cell lines. Considering the well‐documented overexpression of Twist1 in pancreatic cells, we have targeted Twist1 as a therapeutic approach to overcome immune checkpoint resistance. These results contribute to a better understanding of the role of Twist1 in immune checkpoint regulation, particularly its effects on VISTA in pancreatic cancer.

Studies have shown that Twist1 plays a critical role in cell migration and metastasis and high expression of Twist1 is associated with poor clinical outcomes in cancer patients [72, 73]. Twist1 has also been shown to play a key role in the development of chemoresistance [74, 75]. Our data show that the use of the chromatin modifier vorinostat upregulates Twist1 gene expression in pancreatic cancer cell lines. However, the use of this chromatin modifying agent together with the silencing of Twist1 represses the expression of VISTA and the expression of EMT‐related genes. Similar studies show that immune checkpoint molecules induce EMT, which in turn suppresses the activity of cytotoxic T cells, leading to immunotherapy failure [76, 77]. In a study, VISTA expression in breast cancer shows a negative correlation with the epithelial marker E Cadherin and a positive correlation with mesenchymal genes (Vimentin, Slug and Zeb1) [14]. Our findings also indicate that VISTA expression in pancreatic cancer correlates positively with Twist1, suggesting that this mechanism may play an important role in pancreatic cancer progression and sensitivity to immunotherapy.

In conclusion, VISTA has been shown to be closely associated with the development and progression of pancreatic ductal adenocarcinoma for several reasons. VISTA expression in pancreatic adenocarcinoma was extremely high compared to normal tissue in data from GTEx and TCGA. In addition, knockdown of Twist1 led to a significant downregulation of VISTA expression. Furthermore, the combination application of vorinostat, which is commonly used in cancer therapy, with the blockade of Twist1 in pancreatic cancer has substantially downregulated VISTA expression. Our results suggest that VISTA may be a potential target for the treatment of pancreatic cancer. Investigating the ability of HDAC inhibitors to regulate EMT transcription factors and their effects on other immune checkpoints may contribute to the development of new combination strategies for cancer treatment. However, further investigations are needed to clarify the potential impact of the combination therapies of HDAC inhibitors with Twist1 blockade.

## Author Contributions

**Kubra Sena Bas Topcu:** conceptualization (equal), data curation (equal), formal analysis (equal), methodology (equal), project administration (equal), visualization (equal), writing – original draft (equal), writing – review and editing (equal). **Ercan Cacan:** conceptualization (equal), data curation (equal), formal analysis (equal), funding acquisition (equal), investigation (equal), methodology (equal), project administration (equal), resources (equal), supervision (equal), writing – original draft (equal), writing – review and editing (equal).

## Ethics Statement

The authors have nothing to report.

## Conflicts of Interest

The authors declare no conflicts of interest.

## Data Availability

Data available on request from the authors.
